# Childhood tuberculosis: out of sight, out of mind?

**DOI:** 10.1016/j.trstmh.2007.09.011

**Published:** 2008-03

**Authors:** Andrew J. Brent, Suzanne T. Anderson, Beate Kampmann

**Affiliations:** a Wellcome Trust Centre for Research in Clinical Tropical Medicine, Imperial College London, Wright Fleming Institute, Norfolk Place, London W2 1PG, UK; b Academic Department of Paediatrics, Imperial College London, Wright Fleming Institute, Norfolk Place, London W2 1PG, UK

**Keywords:** Tuberculosis, Children, Diagnosis, Epidemiology, DOTS, HIV

## Abstract

Despite significant improvements in tuberculosis (TB) management under the WHO directly observed treatment, short course (DOTS) strategy, childhood TB has been relatively neglected. Children are at high risk of severe disease, and reactivation of latent infection in adulthood perpetuates the epidemic. Almost a million cases of childhood TB are estimated to occur annually, but good-quality epidemiological data are scarce due to inherent difficulties diagnosing paediatric TB. There remains an urgent need both for better diagnostic tests and for robust regional data on the true burden of disease, otherwise childhood TB will remain an essentially ‘invisible’ and therefore neglected disease.

Tuberculosis (TB) has been propelled to the forefront of the international health agenda in the wake of the HIV/AIDS epidemic and the evolution of multidrug-resistant (MDR) and extensively drug-resistant (XDR) strains, stimulating a wide portfolio of research, and injecting new vigour into public health strategies to control the disease. Following the WHO declaration of a global TB emergency in 1993, the directly observed treatment, short course (DOTS) strategy was launched, underpinned by five key elements: sustained political commitment; improved case detection through quality assured bacteriology; standardized short course treatment; assured drug supply; and standardized monitoring and evaluation. Now implemented in nearly 200 countries, DOTS has already led to significant improvements in case detection and treatment outcomes.

Childhood TB, however, has been relatively neglected. The emphasis placed by DOTS on detection of smear-positive cases (which are most infectious) does not address the situation in children, the vast majority of whom have smear-negative disease. In wealthier, low-burden countries, infected children are often identified by contact tracing and given chemoprophylaxis, but limited resources and a much higher disease incidence prohibit this in most high-burden settings. Until recently, most national tuberculosis programmes (NTPs) did not collect data on paediatric TB by age and clinical syndrome, reflecting its perceived lower priority.

TB nevertheless remains an important and potentially preventable cause of childhood illness and death. Young children have a high risk of progression to disease following infection, and are much more likely to develop severe or disseminated TB. Children with latent tuberculosis infection (LTBI) become the reservoir of future disease in adulthood, perpetuating the epidemic. The HIV epidemic, which disproportionately affects young adults, many of whom are co-infected with TB, may also put their children at particularly high risk of infection. Observational data do suggest a disproportionately higher paediatric caseload where deteriorating socioeconomic conditions are accompanied by a higher overall incidence of TB, and children account for more than 20% of TB cases in some high-burden settings.

Worldwide there were nearly a million estimated new cases of childhood TB in 2000 (Nelson and Wells, 2004), but reliable data on the burden of childhood TB remain scarce in most high-burden settings. This is partly due to inherent difficulties in diagnosing paediatric TB. In children the clinical and radiological features of TB overlap those of other common childhood diseases, particularly where HIV and malnutrition (two of the most important risk factors) are endemic. Crucially, a definitive microbiological diagnosis is achieved in only a minority of cases, as young children rarely develop cavitatory lung disease or expectorate sputum, and a greater proportion of cases are extrapulmonary. Diagnosis therefore usually relies on poorly validated clinical case definitions, and both under- and over-diagnosis of paediatric TB are common, with potentially tragic consequences for children who are not diagnosed.

Despite the scale of the problem, progress developing better diagnostics has been slow. Serological assays have so far proved disappointing, and nucleic acid amplification tests for *Mycobacterium tuberculosis* still lack sensitivity; IFN-γ release assays appear to perform better than tuberculin skin testing, but do not differentiate active TB from LTBI (Dinnes et al., 2007). All currently remain too complex and expensive for use at the point of care in resource-poor settings. There remains, therefore, an urgent need for a rapid, reliable and affordable diagnostic test, and it is essential that any such test be properly validated in children. Strategies currently being explored include a urinary antigen test for mycobacterial lipoarabinomannan, the search for disease biomarkers using gene array and proteomic technology, and combinations of existing tests.

Better diagnostics are not the only challenge, however. Other priorities include the need for better, particularly shorter, treatment regimens; improved case management, contact tracing and delivery of chemoprophylaxis in low-resource settings; and the search for a new vaccine. Perhaps most importantly, there is a real need for prospective epidemiological studies to determine the true burden of TB among children in a wide spectrum of settings worldwide. Recent WHO guidance has already taken a significant step in this direction by recommending NTPs record childhood TB cases by age category and clinical syndrome (WHO, 2006). Further high-quality studies underpinned by active case ascertainment and post mortem data are nevertheless required. TB does not currently feature per se in WHO estimates of the causes of death in children (Bryce et al., 2002); yet pneumonia accounts for over 4 million deaths each year in Africa alone, and one necropsy study from Zambia found evidence of TB in nearly 20% of HIV-positive and 26% of HIV-negative children dying of pneumonia (Chintu et al., 2002). As new candidate TB vaccines approach clinical trials, good-quality epidemiological data will be essential to assess their efficacy, cost-effectiveness and optimum vaccine schedules. Above all, without robust regional data on the true burden of disease to inform public health policy, childhood TB will remain an essentially ‘invisible’ and therefore neglected disease.

## Funding

AJB is a Wellcome Trust Training Fellow in Clinical Tropical Medicine. BK is a Wellcome Trust Intermediate Fellow in Clinical Science.

## Conflicts of interest

None declared.

## Ethical approval

Not required.
